# Zebrafish Embryo as an In Vivo Model for Behavioral and Pharmacological Characterization of Methylxanthine Drugs

**DOI:** 10.3390/ijms18030596

**Published:** 2017-03-09

**Authors:** Ram Manohar Basnet, Michela Guarienti, Maurizio Memo

**Affiliations:** Department of Molecular and Translational Medicine, University of Brescia, Viale Europa, 11, 25123 Brescia, Italy; michela.guarienti@unibs.it (M.G.); maurizio.memo@unibs.it (M.M.)

**Keywords:** methylxanthine, zebrafish embryos, behavior, movement, cyclic AMP, Fish Embryo Toxicity test

## Abstract

Zebrafish embryo is emerging as an important tool for behavior analysis as well as toxicity testing. In this study, we compared the effect of nine different methylxanthine drugs using zebrafish embryo as a model. We performed behavioral analysis, biochemical assay and Fish Embryo Toxicity (FET) test in zebrafish embryos after treatment with methylxanthines. Each drug appeared to behave in different ways and showed a distinct pattern of results. Embryos treated with seven out of nine methylxanthines exhibited epileptic-like pattern of movements, the severity of which varied with drugs and doses used. Cyclic AMP measurement showed that, despite of a significant increase in cAMP with some compounds, it was unrelated to the observed movement behavior changes. FET test showed a different pattern of toxicity with different methylxanthines. Each drug could be distinguished from the other based on its effect on mortality, morphological defects and teratogenic effects. In addition, there was a strong positive correlation between the toxic doses (TC_50_) calculated in zebrafish embryos and lethal doses (LD_50_) in rodents obtained from TOXNET database. Taken together, all these findings elucidate the potentiality of zebrafish embryos as an in vivo model for behavioral and toxicity testing of methylxanthines and other related compounds.

## 1. Introduction

Methylxanthines are the methylated derivatives of xanthine. They are heterocyclic organic compounds consisting of pyrimidinedione and imidazole rings coupled with each other [1]. Methylxanthines are phytochemicals found in plants such as coffee, tea, mate, kola nuts and cacao beans [2]. Thus far, seven natural methylxanthines have been identified: aminophylline, caffeine, IBMX, paraxanthine, pentoxifylline, theobromine and theophylline [3]. Plants containing methylxanthines are often used to produce beverages such as coffee, tea, cacao, tejate and several other methylxanthine containing foods, which have been consumed on a daily basis [2]. In particular, caffeine forms a part of very popular and widely consumed soft drinks and energy drinks [4]. A study done in the US population has shown that 87% of the highest consumers of caffeine belong to the age group of 35–64 years. The report of US Department of Agriculture (reported on March 2005) have shown that coffee, carbonated soft drinks and tea accounted for, Respectively, 67%, 27% and 6% of total nonalcoholic beverages consumed in the US [5]. However, the use of methylxanthines is not only limited to beverages. Indeed, they are also being used for other major pharmacological effects [2,6,7].

Caffeine is used as an analeptic and central nervous system stimulant and it is also the most widely consumed psychoactive agent [8]. Theobromine is mainly found in cacao and recent evidence shows that it has great potential as an antitussive agent [9]. Theophylline, aminophylline, doxofylline and diprophylline are used in the therapy of bronchial asthma and chronic obstructive pulmonary diseases (COPD) [8,10,11,12,13]. In particular, doxofylline and diprophylline are two novofyllines synthesized to have fewer side effects than other methylxanthines [11,12,13]. Pentoxifylline is used in the treatment of peripheral vascular diseases and in the management of cerebrovascular insufficiency and diabetic nephropathy [8,10]. Etofylline, another synthetic derivative, is used in lowering the blood lipid level [14]. IBMX has a potential role in ophthalmic diseases and skin tumors [15,16]. In short, these compounds are used in a daily basis, from routine beverages to the treatment of diseases.

One of the major adverse effects of methylxanthines is the potential to cause seizures. The role of methylxanthines in convulsion has long been studied in human and animals [17,18]. These studies have shown that drugs such as aminophylline, caffeine and theophylline can trigger seizures in patients [19]. Therapeutic doses of theophylline exert proconvulsant effects in developing mice and human [20]. Likewise, aminophylline overdose has been shown to induce convulsion in humans and mice [21]. Other methylxanthines, such as IBMX and pentoxifylline, have also proven to have epileptogenic potential in animal studies, although human studies implicating these drugs in epilepsy were not found [22,23]. However, there are several new synthetic derivatives such as etofylline, diprophylline and doxofylline that do not have any proconvulsant capability. In between, some methylxanthines, such as theobromine, show less-evident potential for causing seizures [22].

Rodents are the conventional animal model for behavioral research involving neurotoxic drugs. However, zebrafish embryos are emerging as a model in toxicology and pharmacology for the opportunity to carry out fast reproducible tests and high throughput behavioral screenings [24,25,26,27,28]. The zebrafish genome has approximately 70% homology with that of human and 84% of genes known to be associated with human disease have zebrafish counterparts [29,30]. The small size, rapid external development, optical transparency, less space and husbandry care and easy manipulation are few of the added advantages that play in its favor [31]. Zebrafish possess a wide range of complex behaviors, which can be used to evaluate putative neurotoxic drugs [32,33]. The behavioral characterization of seizure has been done in zebrafish embryos and larvae [34,35]. They have been used as a model for the study of convulsant effect of drugs [36,37]. Seizure-related endpoints such as hyperactivity, twitching, loss of touch-response and immobility are some of the described endpoints of seizures in zebrafish embryos [32,34].

In this study, we used zebrafish embryos to analyze the movement behavior at 24, 48 and 72 h post fertilization (hpf), after treatment with nine different methylxanthine drugs: Aminophylline, Caffeine, Diprophylline, Doxofylline, Etofylline, IBMX, Pentoxifylline, Theobromine and Theophylline. We also measured the level of cyclic aminomonophosphate (cAMP) in whole body extracts of treated embryos to see if it was responsible for the observed behavioral alterations.

One of the advantages of using zebrafish embryos in behavioral studies is they can be used to carry out toxicity tests as well. Fish Embryo Toxicity (FET) test is a standard test recommended by Organization of Economic Cooperation and Development [38] and has an excellent correlation with acute adult fish toxicity tests [39]. We performed FET test with the same nine methylxanthine compounds and a correlation study with mice and rat was then performed.

The effects produced by methylxanthines in the developing embryo might provide us with the insight of its possible effect in the adult fish as well as in higher vertebrates including human. Our aim is to explore the possibility of using zebrafish embryos as an in vivo model to study the behavioral changes and toxicity of methylxanthines and other related methylxanthine compounds.

## 2. Results and Discussion

### 2.1. Behavioral Assay

Movements in vertebrates, such as walking or swimming, depend on the neural networks in the brain and spinal cord [40]. Zebrafish embryos possess a very strong locomotive behavior. The swimming ability of zebrafish can be a useful tool to study the neuromuscular function [41]. Several key time points in the behavioral development of zebrafish have been described. They start spontaneous movement around 19 to 26 hpf. After, they come out of chorionic sac at 48 hpf and start swimming. The motility pattern is initially characterized by burst swimming and slowly changes into beat and glide swimming by the end of 72 hpf [40,42]. These behavioral development time points are crucial as they provide hints about the underlying growth of brain and spinal cord.

To characterize the locomotive behavior of zebrafish embryos after methylxanthine treatment, movement analysis was performed at 24, 48 and 72 hpf. After an initial trial, where each chemical was tested with several increasing concentrations, the dose causing alteration of movements in 50% of treated embryos was selected for each compound to perform the motility test. For diprophylline, the highest concentration (5000 mg/L) was selected, as no movement defects were observed at any tested concentration. The following concentrations were used: aminophylline 500 mg/L, caffeine 150 mg/L, diprophylline 5000 mg/L, doxofylline 1000 mg/L, etofylline 600 mg/L, IBMX 50 mg/L, pentoxifylline 200 mg/L, theobromine 200 mg/L and theophylline 200 mg/L.

**Figure 1:**
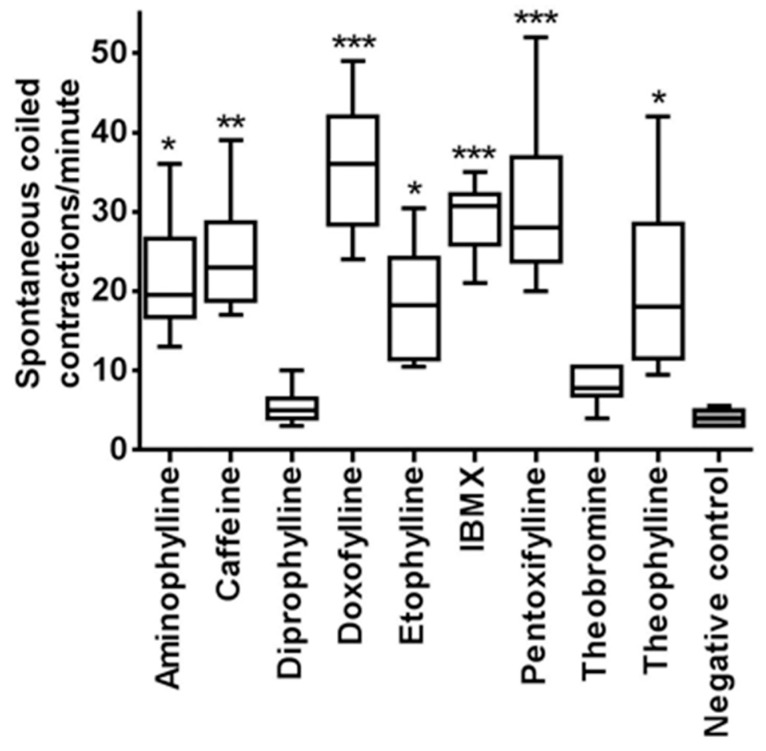
Spontaneous movement quantification at 24 hpf. The following doses of methylxanthine were used: aminophylline 500 mg/L, caffeine 150 mg/L, diprophylline 5000 mg/L, doxofylline 1000 mg/L, etofylline 600 mg/L, IBMX 50 mg/L, pentoxifylline 200 mg/L, theobromine 200 mg/L, and theophylline 200 mg/L. Negative controls were treated with dilution water without any drugs. Data are the mean ± S.D. of three independent experiments. Asterisks indicate statistically significant increase of spontaneous movements compared to negative controls. Significance was determined using ordinary one-way ANOVA, followed by Dunnett’s multiple comparisons test. * *p* < 0.05; ** *p* < 0.005; *** *p* < 0.0001. White columns: methylxanthine treated embryos; grey column: negative controls.

At 24 hpf, comparison of the spontaneous coiling contractions between treated embryos and controls was done. Normally embryos at 24 hpf show a burst of 3–5 coils often followed by period of inactivity that could last >20 s [42]. The number of spontaneous movements of each treated and control embryo was counted for one minute. A box diagram was plotted for each tested compound (Figure 1) and representative time-lapse videos are available as Movies S1–S3. Results were analyzed by one-way ANOVA followed by Dunnett’s multiple comparisons test. Spontaneous head and tail coils of zebrafish embryos were significantly increased after 24 h of treatment with methylxanthines as compared to controls, with the exception of theobromine and diprophylline. IBMX, pentoxifylline and doxofylline caused the higher number of coiling contractions in the embryos. Caffeine, theophylline, etofylline and aminophylline also caused this kind of movements, although their number was less pronounced. However, with theobromine and diprophylline treated embryos, the movement was normal.

**Figure 2:**
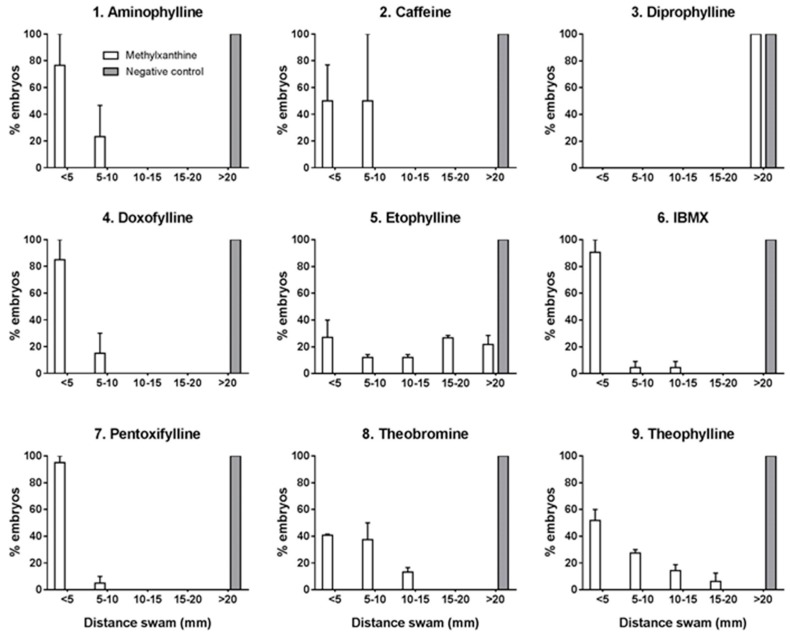
Touch-and-response test performed at 72 hpf. *X*-axis shows the distance swam by the embryos, *Y*-axis shows the percentage of embryos that swam different distances. The following doses of methylxanthine were used: aminophylline 500 mg/L, caffeine 150 mg/L, diprophylline 5000 mg/L, doxofylline 1000 mg/L, etofylline 600 mg/L, IBMX 50 mg/L, pentoxifylline 200 mg/L, theobromine 200 mg/L, and theophylline 200 mg/L. Negative controls were treated with dilution water without any drugs. Data are the mean ± S.D. of three independent experiments. White columns: methylxanthine treated embryos; grey columns: negative controls.

At 48 and 72 hpf, touch-evoked movement test was performed following the procedure as described previously, with some modifications [43]. The test was performed on groups of 20 embryos for each methylxanthine compound and control. Each embryo from treated and control group was placed at the center of a motility wheel containing four concentric circles (5, 10, 15 and 20 mm Ø). The embryo was then touched gently in the tail with embryo poker and the distance swam in the concentric circles was recorded and categorized into five groups: <5, 5–10, 10–15, 15–20 and >20 mm. Results were then converted into percentage following which descriptive statistical analysis was performed and the results were represented with histogram (Figure 2). The test showed a compromised motility at 48 and 72 hpf in treated embryos, with the exception of diprophylline. Treated embryos showed diverse pattern, most commonly dragging and round the circle movement. The swim speed was also slowed in these embryos, with less distance covered in one episode. Burst swimming, normally present in control embryos at 48 and 72 hpf, was absent in treated ones. In particular, after 72 hpf, there was absence of beat and glide swimming seen in controls. Some developmental defects observed in treated embryos at 48 and 72 hpf, such as reduced detachment of tail, might have affected the results obtained in the touch-and-response test.

Methylxanthines show the tendency to cause seizures in humans and animals [19]. The increased number of spontaneous movements at 24 hpf in fish embryos can be categorized as hyperactivity and twitching, which are two types of epileptic movements [34,35,44]. Out of the nine compounds we tested, seven induced epileptic-like movement in zebrafish embryos at 24 hpf. After 48 h of treatment, the swimming was impaired and this progressed further at 72 hpf. Our finding of epileptic-like movements during the initial phase followed by impaired movement resembled a previous study with convulsant drugs in larvae and adult zebrafish. Indeed, they have shown similar pattern of increased movement during the initial period of treatment and paralyzed movement after prolonged exposure [34]. Therefore, our study showed that zebrafish embryo could be a good animal model to study complex behavioral pattern induced by a class of neurotoxic drugs, such as methylxanthines.

### 2.2. Cyclic AMP Measurement

**Figure 3:**
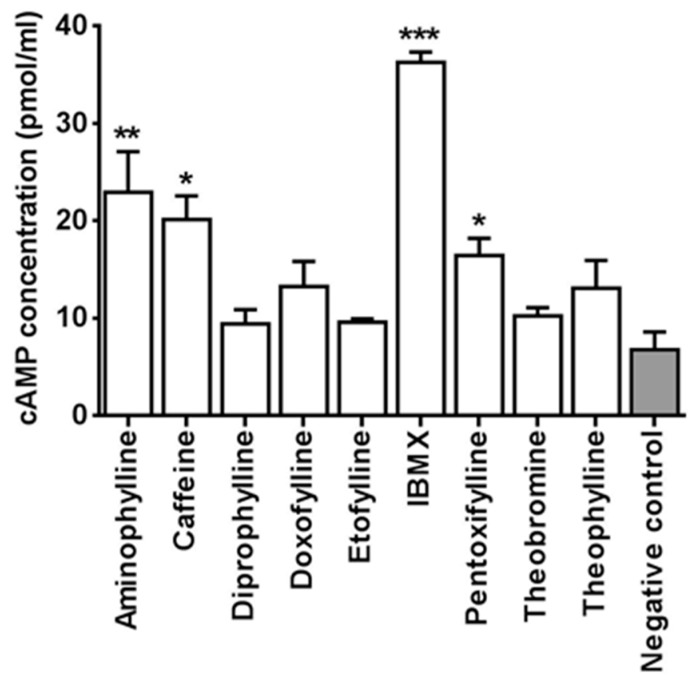
Cyclic AMP level in whole zebrafish embryos extract. The following doses of methylxanthine were used: aminophylline 500 mg/L, caffeine 150 mg/L, diprophylline 5000 mg/L, doxofylline 1000 mg/L, etofylline 600 mg/L, IBMX 50 mg/L, pentoxifylline 200 mg/L, theobromine 200 mg/L, and theophylline 200 mg/L. Negative controls were treated with dilution water without any drugs. Data are the mean ± S.D. of three independent experiments. Asterisks indicate statistically significant increase of cAMP compared to negative controls. Significance was determined using ordinary one-way ANOVA, followed by Dunnett’s multiple comparisons test. * *p* < 0.05; ** *p* < 0.005; *** *p* < 0.0001. White columns: methylxanthine treated embryos; grey column: negative controls.

One of the major mechanisms of action of methylxanthine is inhibition of phosphodiesterase enzyme and this results in increased cellular cyclic AMP (cAMP) [6]. Thus, we investigated if cAMP pathway is involved in the locomotor alterations and toxicity seen in treated embryos. The cAMP was measured by ELISA immunoassay in whole zebrafish embryo extracts. The embryos used for this test were treated till 48 hpf with the same concentrations of methylxanthine used for the motility test. The results showed a relative increase in cAMP levels in embryos treated with IBMX, caffeine, aminophylline, pentoxifylline, theophylline, and doxofylline (Figure 3). One-way ANOVA with Dunnett’s multiple comparison test showed that the increase in cAMP level was statistically significant in embryos treated respectively with IBMX (*p* < 0.0001), aminophylline (*p* < 0.005), caffeine and pentoxifylline (*p* < 0.05). The ratio of each compound as compared to the baseline control was also calculated by using the mean values of cAMP quantification. The increase in cAMP was the highest with IBMX (>6 times the baseline control). It was about three times the baseline with caffeine and aminophylline and about two times the baseline with pentoxifylline, theophylline and doxofylline.

The increase in cAMP with most of the methylxanthines treated embryos in our study showed a plausible evidence of involvement of similar cAMP pathway in the toxicity of zebrafish and higher vertebrates. Methylxanthines increases cAMP by inhibiting the phosphodiesterase enzymes (PDEs), however not all the methylxanthines act by this way. The increase in cyclic AMP also depends on the dose of the drugs used and their potency [2,6]. In our study, some drugs such as IBMX, caffeine, theophylline and pentoxifylline showed a significant increase in cyclic AMP in zebrafish embryos. Of special note was that of IBMX, which showed a substantial increase in cAMP as compared to all the other drugs. Study in rat have shown that IBMX increases cAMP not only by inhibiting the PDEs but also by stimulating the adenylate cyclase via blocking the function of the regulatory protein G_i_. Thus, the marked increase of cAMP with IBMX treated embryos could be attributed to its action as stimulant of adenylate cyclase in addition to its inhibitory effect on PDEs [45]. Compounds such as diprophylline, theobromine and etofylline, which did not cause much increase in cAMP in the zebrafish embryos, are shown to be less potent than other methylxanthines in higher vertebrates studies as well. An investigation done in guinea pig have shown that diprophylline was five times less effective in inhibiting the tracheal PDE, however, it was found to be virtually ineffective in increasing the cAMP [46]. Similarly, compounds such as theobromine and etofylline, which did not cause much increase in cAMP in the zebrafish embryos, are shown to be less potent than other methylxanthines in other higher animal studies as well [47]. In our study, the increase in cAMP may be involved in the toxicity of the embryos treated with IBMX, aminophylline, caffeine and pentoxifylline However, we also found that some drugs such as theobromine, etofylline, doxofylline and theophylline had shown toxic effects and behavioral alterations in the embryos irrespective of the increase in cAMP. Therefore, it could not be said with certainty that the increase in cAMP is responsible for the behavioral modifications and toxic effects observed in treated embryos.

### 2.3. Fish Embryo Toxicity (FET) Test

A Fish Embryo Toxicity (FET) test was performed to verify if other specific developmental endpoints, in addition to locomotor alteration, were affected by methylxanthine treatment. Preliminary experiments were carried out to set the optimal concentration for each compound to be used in zebrafish embryos (data not shown). After the initial trial, five increasing concentrations of each compound were selected (Table 1) to perform the FET test as described [39].

Three aspects of toxicity, namely mortality, morphological developmental defects and teratogenic effects, were evaluated and scored in embryos at 24, 48 and 72 hpf as previously described [48]. Briefly, specific developmental hallmarks were examined in order to identify any morphological defect: tail detachment, somite formation, eye development, movement and heartbeat at 24 hpf; in addition also blood circulation and pigmentation were evaluated at 48 hpf and pectoral fin development, mouth protrusion and hatching from the chorion at 72 hpf. Embryos with morphological defects received higher score, so at the end of the test significant developmental anomalies corresponded to high General Morphological defect Score (GMS). Teratogenic effects were assessed in each treated embryo as the absence (0) or presence (1) of any malformations from head to tail region: head, eye, ear, heart, yolk, trunk and tail. At the end of the FET test, the General Teratogenic effect Score (GTS) was directly proportional to the teratogenicity of the compound under examination.

**Figure 4:**
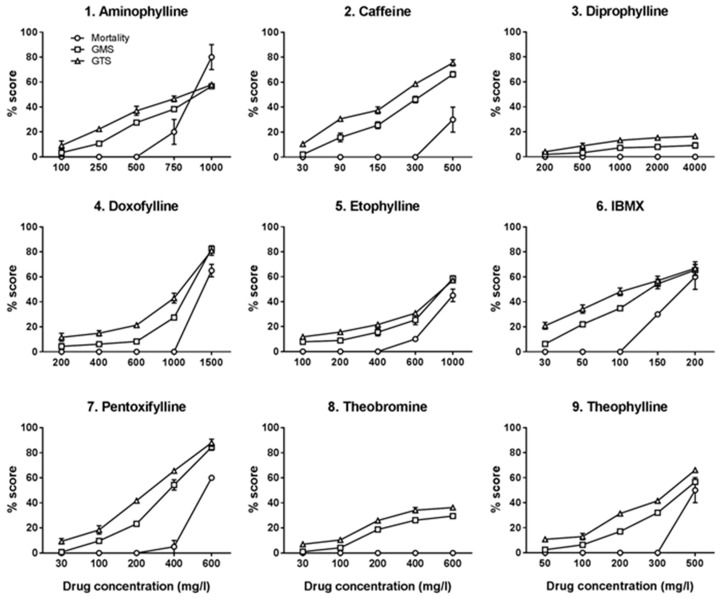
Effect of nine methylxanthine compounds on zebrafish embryos development. *X*-axis shows the five increasing concentrations of each methylxanthine drug used in the FET test. Data are the mean ± S.D. of three independent experiments. (GMS) General Morphological defects Score; (GTS) General Teratogenicity Score. White circles: mortality rate; white squares: GMS; white triangles: GTS.

Mortality score, GMS and GTS were obtained for each tested concentration of the nine methylxanthine compounds. These scores, from three independent experiments, were averaged, converted into percentage and used to plot a drug–response curve for each of the nine methylxanthine compounds (Figure 4). The results showed that, barring diprophylline, all the tested compounds caused varying degrees of dose dependent toxicity and lethality.

The concentrations causing 50% of the maximal toxic effect (TC_50_) for mortality, GMS and GTS were calculated for each compound by linear interpolation and extrapolation (Table 2). Of note, IBMX was the most toxic compound, with the lower TC_50_ (TC_50_ for mortality = 183.5 mg/L; TC_50_ for GMS = 139 mg/L and TC_50_ for GTS = 111 mg/L), whereas diprophylline was the least toxic with TC_50_ far greater than the highest concentration used (4000 mg/L). A discrepancy between the toxicity and lethality of caffeine and theobromine was observed. Caffeine showed dose dependent toxicity with a disproportionately lower lethality (TC_50_ for mortality = 634 mg/L; TC_50_ for GMS = 329 mg/L and GTS = 251 mg/L). Theobromine did not caused any mortality with the highest concentration tested (600 mg/L), however it produced dose dependent toxicity at morphological and teratogenic level (TC_50_ for GMS = 1840 mg/L and TC_50_ for GTS = 1975 mg/L).

We also found a similar pattern in the severity of behavioral defect and toxicity in the embryos. Indeed, drugs with more severe movement defects showed higher toxicity in FET test and vice versa. For instance, IBMX which was the most toxic compound, also showed a higher movement defect; diprophylline, which was virtually non toxic, did not show any movement defect in the highest tested concentration. The following concentrations of methylxanthine drugs were used to perform behavioral tests: aminophylline 500 mg/L, caffeine 150 mg/L, diprophylline 5000 mg/L, doxofylline 1000 mg/L, etofylline 600 mg/L, IBMX 50 mg/L, pentoxifylline 200 mg/L, theobromine 200 mg/L and theophylline 200 mg/L. The doses employed in the movement assay corresponded to doses that gave approximately 30% morphological alterations in the FET test. However, the majority of the morphological alterations observed in the FET test started appearing only after 48 hpf and only negligible morphological alterations were present at 24 hpf. Thus, the increase in spontaneous head and tail coiling contractions at 24 hpf can be considered independent of the morphological defects. At 48 and 72 hpf, the onset of noticeable morphological alterations might have aggravated the movement defects observed at that stage of development.

Analysis of individual endpoints concerning GMS and GTS showed that a similar pattern of morphological defect and teratogenic effect was observed among the methylxanthine treated embryos. Morphological endpoints most commonly affected, in addition to altered movements, were irregular somite formation and abnormal heart beating and blood circulation. Teratogenic effects most commonly observed were yolk sac deformation and/or edema, pericardial edema, malformation of tail and scoliosis. The specific endpoints affected by these compounds are tabulated in Table 3 and Table S1.

Analysis of dose–response curve for mortality, GMS and GTS showed a peculiar pattern of toxicity with each methylxanthine compound. For instance, IBMX was toxic even in the lower doses, affecting almost equally all three endpoints. Other drugs, such as diprophylline, did not produce any toxicity even with larger doses. Of note, the pattern of some methylxanthines, such as caffeine and theobromine, pointed out high morphological and teratogenicity defects with less pronounced mortality. Doxophylline showed a safety threshold within which there were no mortality and low morphological and teratogenic defects and passed which it produced very high toxic effects. Overall, these results showed that each methylxanthines has its own peculiar pattern of toxicity based on mortality, GMS and GTS assessment and these could be studied with zebrafish embryos as a model. Indeed, currently zebrafish embryos have found their increasing use as a model for drug toxicity assessment of diverse group of compounds and our results showed that FET test with added endpoints of GMS and GTS could also be utilized for this purpose [49]. Our findings were consistent with the available data from higher animals such as mice and human. As in mice, IBMX was the most toxic compound and diprophylline the least toxic one in zebrafish embryos among the nine tested methylxanthine. Some compounds such as doxofylline and diprophylline, which were developed to reduce the adverse effects of theophylline in treatment of COPD, were less toxic in zebrafish embryos as well [50]. Similarly, theobromine, which was clinically found to be 10 times less potent than caffeine, was approximately 6.5 times less potent in zebrafish embryos [51,52,53].

### 2.4. Correlation between TC_50_ Zebrafish and LD_50_ Mice and Rat

Correlation and linear regression analyses were done to see if toxicity of the nine tested methylxanthines observed in zebrafish were comparable to that in rodents, in this case mice and rat. The toxic concentration at which embryos showed 50% of mortality, general morphological defect and general teratogenic effect (TC_50_ for mortality, GMS and GTS, respectively) was calculated for each compound by linear interpolation and extrapolation after the FET test (Table 2). The lethal dose of the nine compounds producing 50% mortality in rodents (LD_50_) were obtained from “ChemIDplus” (available on: https://chem.sis.nlm.nih.gov/chemidplus/chemidlite.jsp) [54], a Toxicology Data Network (TOXNET) database (Table 4). The correlation analysis were performed by using the LD_50_ values of each methylxanthine compound in mice and rat obtained from “ChemIDplus” and TC_50_ values calculated in zebrafish embryos after the FET test.

**Figure 5:**
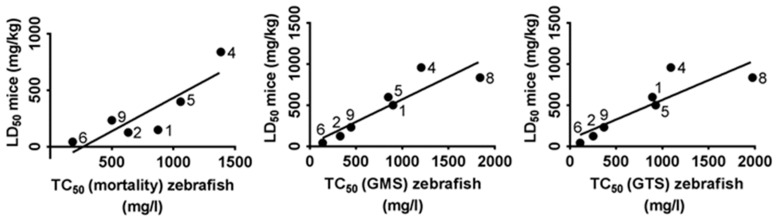
Correlation between TC_50_ (Toxic Concentration, 50%) of zebrafish (*X*-axis) and LD_50_ (Lethal Dose, 50%) of mice (*Y*-axis). The analysis included seven of the nine tested methylxanthines: diprophylline was excluded because the value was too large and pentoxifylline because it was an outlier. Numbers linked to each dot correspond to a methylxanthine compound, as reported in Table 2.

For the correlation analysis between mice and zebrafish embryos, diprophylline and pentoxifylline were excluded from the study. We could not obtain the TC_50_ value of diprophylline in zebrafish, since the dose–response curves were virtually flat and there were minimal toxic effects (<20%) even at the highest concentration tested. Thus, diprophylline was excluded from the analysis. When performed with eight out of nine compounds, the correlation resulted insignificant both for mortality, GMS and GTS. This might be due to the high toxicity of pentoxifylline observed in zebrafish as compared to mice. When pentoxifylline was excluded from the study, the correlation analysis showed that LD_50_ in mice was strongly correlated with TC_50_ in zebrafish, both for mortality (*r* = 0.86, *p* < 0.05), GMS (*r* = 0.85, *p* < 0.05) and GTS (*r* = 0.85, *p* < 0.05), respectively. Results are represented in Figure 5. Linear regression analysis also showed that the correlation between the TC_50_ in zebrafish and LD_50_ in mice is linear (*r*^2^ = 0.71, *p* < 0.05 for mortality, *r*^2^ = 0.71, *p* < 0.05 for GMS and *r*^2^ = 0.63, *p* < 0.05 for GTS, respectively).

**Figure 6:**
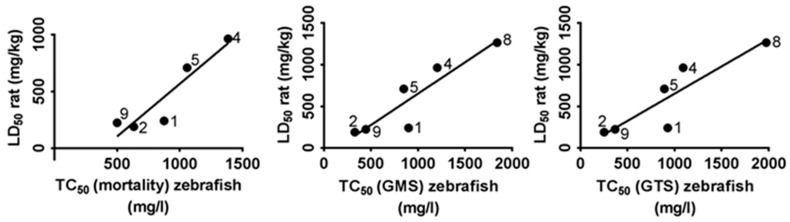
Correlation between TC_50_ (Toxic Concentration, 50%) of zebrafish (*X*-axis) and LD_50_ (Lethal Dose, 50%) of rat (*Y*-axis). The analysis included six of the nine tested methylxanthines: diprophylline was excluded because the value was too large and pentoxifylline because it was an outlier; no data were available about IBMX toxicity in rat. Numbers linked to each dot correspond to a methylxanthine compound, as reported in Table 2.

For the correlation analysis between rat and zebrafish embryos diprophylline and pentoxifylline were excluded from the study because of the reasons mentioned above. IBMX was also excluded from the study due to the unavailability of toxicity data in rat. Thus, the analysis in rat included six out of nine tested methylxanthine compounds. The analyses, represented in Figure 6, showed that LD_50_ in rat was strongly correlated with TC_50_ in zebrafish for mortality (*r* = 0.92, *p* < 0.05), GMS (*r* = 0.94; *p* < 0.05) and GTS (*r* = 0.94; *p* < 0.05). Linear regression analysis also showed that the correlation between TC_50_ in zebrafish and LD_50_ in rat was linear (*r*^2^ = 0.85 *p* < 0.05 for mortality, *r*^2^ = 0.83; *p* < 0.05 for GMS and *r^2^* = 0.79; *p* < 0.05 for GTS, respectively).

Correlation and linear regression analysis showed that toxicity of methylxanthines correlated well in rodents and zebrafish embryos. There are several studies in which zebrafish embryos are already employed as a model for toxicity screening. Indeed several researches have demonstrated the effectiveness of FET test to predict acute toxicity of chemicals. In particular these studies underline the important possibility to implement the validity of FET test results by adding additional endpoints, such as behavioral tests [51,64,65,66,67]. The correlation of the TC_50_ calculated in zebrafish with that available of rodents opened up the possibility of using zebrafish embryo as a model for toxicity testing and screening of methylxanthines and other related compounds. Our results are further boosted from the results of comparative study done between adult and embryo zebrafish toxicity tests, which showed qualitatively similar data [39,68].

## 3. Materials and Methods

### 3.1. Zebrafish Maintenance and Egg Collection

All animals were handled according to national and international animal care guidelines. Current Italian rules do not require approval for research on zebrafish embryos.

A group of 50 healthy adult wild type zebrafish (AB strain) from the DMMT facility of the University of Brescia were used for egg production. Fishes were raised and maintained under standard laboratory conditions as described, at 27 ± 1 °C on a 14 h light:10 h dark cycle [69]. Fishes were fed thrice a day with a combination of granular dry food and fresh artemia (both purchased from Special Diet Services, SDS Diets). Nine months old male and female zebrafish were put in the breeding tank overnight in a 1:2 ratio and the eggs were harvested next morning, immediately after spawning. The collected eggs were placed in 10 cm Ø Petri dishes in fish water with methylene blue (0.1 g/L Instant Ocean Salt; 0.1 g/L NaHCO3; 0.19 g/L CaSO_4_; methylene blue 0.5 ppm in sterile deionized water). Eggs were visualized under Leica MZ16F stereomicroscope (Leica Microsystems Ltd., Cambridge, UK) and any unfertilized zygote, dead embryo or eggs at advanced stages of development were discarded.

### 3.2. Behavioral Assay

Zebrafish embryos were treated with the following nine methylxanthine compounds: Aminophylline (#A1755, CAS 317-34-0, solubility in H_2_O 50 mg/mL), Caffeine (#C0750, CAS 58-08-2, solubility in H_2_O 15 mg/mL), Diprophylline (#D0633, CAS 479-18-5, solubility in H_2_O 50 mg/mL), Etofylline (#H9006, CAS 519-37-9, solubility in H_2_O 100 mg/mL), IBMX (#I5879, CAS 28822-58-4, solubility in H_2_O 22.225 mg/mL), Theobromine (#T4500, CAS 83-67-0, solubility in H_2_O 5 mg/mL) and Theophylline (#T1633, CAS 58-55-9, solubility in H_2_O 8.3 mg/mL) from Sigma-Aldrich (Milan, Italy); Doxofylline (#Ab142296, CAS 69975-86-6, solubility in H_2_O 2 mg/mL) and Pentoxifylline (#Ab120725, CAS 6493-05-6, solubility in H_2_O 43 mg/mL) from Abcam (Cambridge, UK).

An initial trial was performed, testing each chemical with several increasing concentrations. All the chemicals were dissolved in sterile deionized water. Embryos incubated in dilution water were used as negative controls. Within 1.5 hpf fertilized eggs were randomly distributed in 10 cm Ø Petri dishes containing 10 mL of the different concentrations of the test solutions or negative control (about 40 embryos per plate). Embryos were kept at 27 ± 1 °C until reaching the correct stage of development to carry out the test. After the preliminary assays, the dose causing alteration of movements in 50% of treated embryos was selected for each compound to perform the motility test. For diprophylline, the highest concentration (5000 mg/L) was selected, as no movement defects were observed at any tested concentration. The following concentrations were used: aminophylline 500 mg/L, caffeine 150 mg/L, diprophylline 5000 mg/L, doxofylline 1000 mg/L, etofylline 600 mg/L, IBMX 50 mg/L, pentoxifylline 200 mg/L, theobromine 200 mg/L and theophylline 200 mg/L.

At 24 hpf, ten embryos for each compound were observed for head-tail coil spontaneous contractions for one minute under Leica MZ16F stereomicroscope (Leica Microsystems Ltd.). The total number of head-tail coil movements of three independent experiments was recorded for each embryo, the average was calculated and a box diagram was plotted for treated and control embryos. One-way ANOVA followed by Dunnett’s multiple comparisons test was done to see any significance in the movement between treated and control embryos.

At 48 and 72 hpf, 20 embryos for each compound were subjected to touch-and-response test, following a modified previously described protocol [43]. A motility wheel, consisting in four concentric circles of respectively 5, 10, 15, 20 mm diameter, was placed on the microscope stage and centered at the bottom of a 60 mm Ø Petri dish containing fish water. A single embryo was transferred in the middle of the concentric circles and when it was stationary in the center of the plate the test could start. Each embryo was gently touched at tail with an embryo poker and the distance it swam in the predetermined concentric circles was recorded. If the embryo did not completely exit the first designated circle, it was repositioned in the center of the plate and the test was repeated. If the embryo could not cross the first circle after multiple attempts (5 attempts), it was determined as incapable of exiting the circle. Once data of the distance swam by all 20 embryos treated by each drug and negative control were obtained, the percentage of embryos crossing each predetermined concentric circle were calculated. The test was performed three times independently and results were represented in a bar diagram.

### 3.3. Measurement of Cyclic AMP (cAMP)

Whole body cAMP was measured using Cyclic AMP Direct ELISA kit (Abcam). Embryos were treated with methylxanthine compounds from 8 to 16 cell stage until 48 hpf. The concentrations used were the same as that in the motility test.

At 48 hpf, 40 embryos for each compound were collected in an Eppendorf tube, washed with sterile water and stored O.N. at −80 °C without water. Then embryos were homogenized with 100 μL 0.1 M HCl and centrifuged at 600 rpm for 10 min at 40 °C. Supernatant was collected and used directly for ELISA assay following the manufacturer’s instruction. The optical density was measured at 405 nm using Ensight plate reader (PerkinElmer, Waltham, MA, USA). The test was done three times independently. One-way ANOVA followed by Dunnett’s multiple comparisons test was done to see any significant difference in cAMP between treated and control embryos.

### 3.4. Fish Embryo Toxicity (FET) Test

#### 3.4.1. Embryo Exposure

Zebrafish embryos were treated with the same nine methylxanthine used in motility test dissolved in sterile deionized water. Dilution water was used as negative control (embryos mortality <10%), while 3,4-dichloroaniline from Sigma-Aldrich (3.7 mg/L) in sterile deionized water was used as positive control (embryos mortality >10%) [39].

An initial trial was performed, testing each chemical with several increasing concentrations in the wide range of 5–2000 mg/L. The final concentrations were determined based on the GMS and GTS values obtained in this initial trial. The lowest concentration was determined so that the embryos had minimal effect (<10% GMS and GTS scores) and the highest concentration was selected so that the embryos had at least >50% morphological defects. With diprophylline, we tested till 5000 mg/L but we did not find any significant rise in GMS and GTS. After that, five different concentrations of each compound were selected (Table 1) to perform the FET test as described [39,48]. Briefly, within 1 hpf fertilized eggs were randomly distributed in 5 cm Ø Petri dishes containing 5 mL of the different concentrations of the test solutions and negative or positive controls (about 25 embryos per plate). Then, embryos between 8 and 16 cell stage (1.25 and 1.5 hpf) were selected under a Leica MZ16F stereomicroscope (Leica Microsystems Ltd.) and transferred in each well of 24-well plates containing 2 mL of test or control solutions. Ten embryos for each concentration were used, so a total amount of 50 embryos for each methylxanthine compound were analyzed. All the FET tests were performed in triplicate and the exposure was static to guarantee the minimum manipulation of the embryos.

#### 3.4.2. Evaluation of Embryos

Embryos were evaluated in a semi-quantitative way at 24, 48 and 72 hpf under Leica MZ16F stereomicroscope (Leica Microsystems Ltd.), as described [48]. At each time point, the mortality rate was recorded as present or absent, while morphological defects and teratogenic effects were evaluated and scored on the basis of a scoring matrix [48]. Of note, chorions were not manually removed, to guarantee minimum manipulation of embryos. Moreover, the natural ability to hatch from the chorion after 48 hpf was one of the developmental hallmark evaluated in the scoring matrix. At the end of the test a General Morphological defect Score (GMS) and General Teratogenic effect Score (GTS) were obtained. The scores of three independent experiments were converted into percentage and used to plot a drug–response curve for each of the nine methylxanthine compounds.

Linear interpolation and extrapolation allowed calculating the TC_50_, i.e., the toxic concentration at which there was 50% of dead embryos (TC_50_ for mortality), 50% of morphological defects (TC_50_ for GMS) and 50% of teratogenic effects (TC_50_ for GTS).

Morphological and teratogenic endpoints were also analyzed individually to investigate if specific features were affected by methylxanthine exposure. For this, a table was prepared listing the major affected endpoints of GMS and GTS. The affected endpoint is represented as “+” and unaffected endpoint is represented as “−”. The cut off for an endpoint to be listed “+” was ≥50% defect of a parameter by at least one of the test concentration in FET test.

### 3.5. Correlation between TC_50_ Zebrafish and LD_50_ Rodent

The toxic concentration at which embryos showed 50% of mortality, general morphological defect and general teratogenic effect (TC_50_ for mortality, GMS and GTS, respectively) was compared with that of lethal doses (LD_50_) of mice and rat. Through the search in the ChemID*p*lus (TOXNET database) [54], we obtained the LD_50_ in rodents for all the tested methylxanthine compounds (Table 4). We used LD_50_ concerning the oral administration in mice and rat for comparison with zebrafish TC_50_ data, except with IBMX, the LD_50_ of which is available only in intraperitoneal route.

### 3.6 Statistical Analysis

Statistical analysis were made using GraphPad Prism 6.01 version (GraphPad Software, La Jolla, CA, USA). D’Agostino and Pearson omnibus normality test was performed to verify normal distribution of data. Then analysis of variance (one-way ANOVA) followed by Dunnett’s test was performed to evaluate significant differences between the groups of data.

## 4. Conclusions

In the present study, zebrafish embryos were used to assess their feasibility as an in vivo animal model for the behavioral and toxicity testing of methylxanthines. The effect of nine different methylxanthine compounds was evaluated by behavioral analysis, biochemical assay and FET test in zebrafish embryos.

Movement analysis showed that developmental milestones of zebrafish embryo can be utilized to perform behavioral analysis. We documented an increase in spontaneous head and tail coil movements in treated zebrafish embryos. These spontaneous coils contractions were associated with epileptic-like movement features described in zebrafish, such as twitching, jittery and tremor. However, these movement defects could have been due to the morphological abnormalities observed especially at 48 and 72 hpf.

FET test showed a peculiar pattern of toxicity with each compound, primarily affecting movement and heart beating. Each drug could be distinguished from the other based on the effect on mortality, GMS and GTS. The tested methylxanthine exhibited a wide range of potency, with IBMX as the most potent compound and diprophylline the least. This pattern also showed a great correlation with the toxicity studies from rodents. Indeed, the strong correlation between the toxicity values obtained with zebrafish embryos with the already available rat and mice toxicity data showed that zebrafish embryos can be used as an alternative model for the toxicity studies of methylxanthines.

However, biochemical analysis showed that despite of the significant increase in cAMP with few of the compounds, this was unrelated to the behavioral alterations and toxicity produced by these compounds.

To conclude, our findings confluence to emphasize the potential of zebrafish embryos to study behavioral alterations and toxic effects of methylxanthines. It could also act as a model for the screening of other related methylxanthines compound for any potential epileptogenic effects and toxicity.

## Figures and Tables

**Table 1:** The five doses of each methylxanthine compound used in the FET test.

Compound	Dose (mg/L)				
Aminophylline	100	250	500	750	1000
Caffeine	30	60	90	150	300
Diprophylline	200	500	1000	2000	4000
Doxofylline	200	400	600	1000	1500
Etofylline	100	200	400	600	1000
IBMX	30	50	100	150	200
Pentoxifylline	30	100	200	400	600
Theobromine	30	100	200	400	600
Theophylline	50	100	200	300	500

**Table 2:** TC_50_ Mortality, TC_50_ GMS and TC_50_ GTS of the nine methylxanthine compounds tested on zebrafish embryos in the FET test.

Methylxanthine Compound	TC_50_ Mortality	TC_50_ GMS	TC_50_ GTS
	mg/L		
1. Aminophylline	875	900	827
2. Caffeine	634	329	251
3. Diprophylline	*	*	*
4. Doxofylline	1385	1205	1092
5. Etophylline	1058	848	892
6. IBMX	183.5	139	111
7. Pentoxifylline	564	372	243
8. Theobromine	**	1840	1975
9. Theophylline	500	446	369

* The TC_50_ could not be calculated for diprophylline as the dose–response curve was virtually flat. ** Theobromine did not show any mortality at the highest concentration tested, so the TC_50_ could not be calculated.

**Table 3:** Specific developmental and teratogenicity endpoints affected by methylxanthines.

Affected Endpoints		Control	Aminophylline Caffeine Doxofylline IBMX Pentoxifylline Theobromine Theophylline	Diprophylline	Etofylline
Morphological endpoints	Somite formation	−	+	−	+
	Movement defect	−	+	−	+
	Abnormal heart beating	−	+	+	+
	Abnormal blood circulation	−	+	−	+
Teratogenic endpoints	Yolk sac edema and/or deformation	−	+	−	+
	Pericardial edema	−	+	−	+
	Malformation tail	−	+	−	+
	Scoliosis	−	+	−	−

+: indicates the endpoint is affected; −: indicates the endpoint is not affected.

**Table 4:** LD_50_ values of methylxanthine compounds in mice and rat available in literature and the respective source of data.

Compounds	LD_50_ (mg/kg) Mice	LD_50_ (mg/kg) Rat	References
Aminophylline	150	243	[55]
Caffeine	127	192	[56]
Diprophylline	1954	>400	[57]
Doxofylline	841	965	[58]
Etophylline	400	710	[59]
IBMX	44	-	[60]
Pentoxifylline	1225	1170	[61]
Theobromine	837	1265	[62]
Theophylline	235	225	[63]

All data refer to oral administration, except with LD_50_ of IBMX which is available only in intraperitoneal route in mice.

## Supplementary Materials

Supplementary materials can be found at www.mdpi.com/1422-0067/18/3/596/s1.

## References

[1] Talik P., Krzek J., Ekiert R.J. (2012). Analytical techniques used for determination of methylxanthines and their analogues—Recent advances. Sep. Purif. Rev..

[2] Monteiro J., Alves M., Oliveira P., Silva B. (2016). Structure-bioactivity relationships of methylxanthines: Trying to make sense of all the promises and the drawbacks. Molecules.

[3] Oñatibia-Astibia A., Martínez-Pinilla E., Franco R. (2016). The potential of methylxanthine-based therapies in pediatric respiratory tract diseases. Respir. Med..

[4] Frary C.D., Johnson R.K., Wang M.Q. (2005). Food sources and intakes of caffeine in the diets of persons in the United States. J. Am. Diet. Assoc..

[5] US Department of Agriculture Economic Research Service. Food Contributions of Nonalcoholic Beverages to the U.S. Diet. https://www.ers.usda.gov/publications/pub-details/?pubid=44713.

[6] Franco R., Oñatibia-Astibia A., Martínez-Pinilla E. (2013). Health benefits of methylxanthines in cacao and chocolate. Nutrients.

[7] Sotelo A., Soleri D., Wacher C., Sánchez-Chinchillas A., Argote R.M. (2012). Chemical and nutritional composition of tejate, a traditional maize and cacao beverage from the central valleys of oaxaca, mexico. Plant Foods Hum. Nutr..

[8] Szentmiklósi A.J., Cseppentō A., Gesztelyi R., Zsuga J., Körtvély A., Harmati G., Nánási P.P. (2011). Xanthine derivatives in the heart: Blessed or cursed?. Curr. Med. Chem..

[9] Tilley S.L. (2011). Methylxanthines in asthma. Handb. Exp. Pharmacol..

[10] McCarty M.F., O’Keefe J.H., DiNicolantonio J.J. (2016). Pentoxifylline for vascular health: A brief review of the literature. Open Heart.

[11] Stablein J.J., Samaan S.S., Bukantz S.C., Lockey R.F. (1983). Pharmacokinetics and bioavailability of three dyphylline preparations. Eur. J. Clin. Pharmacol..

[12] Riffo-Vasquez Y., Man F., Page C.P. (2014). Doxofylline, a novofylline inhibits lung inflammation induced by lipopolysacharide in the mouse. Pulm. Pharmacol. Ther..

[13] Page C.P. (2010). Doxofylline: A “novofylline”. Pulm. Pharmacol. Ther..

[14] Ujházy E., Onderová E., Horáková M., Bencová E., Durisová M., Nosál R., Balonová T., Zeljenková D. (1989). Teratological study of the hypolipidaemic drugs etofylline clofibrate (VULM) and fenofibrate in swiss mice. Pharmacol. Toxicol..

[15] Perchdlet J., Boutwell R.K. (1982). Comparison of the effects of 3-isobutyl-l-methylxanthine and adenosine cyclic 3′:5′-monophosphate on the induction of skin tumors by the initiation-promotion protocol and by the complete carcinogenesis process. Carcinogenesis.

[16] Gilbard J.P. (1994). Treatment of keratoconjunctivitis sicca in rabbits with 3-isobutyl-1-methylxanthine. Arch. Ophthalmol..

[17] Zwillich C.W., Sutton F.D., Neff T.A., Cohn W.M., Matthay R.A., Weinberger M.M. (1975). Theophylline-induced seizures in adults. Correlation with serum concentrations. Ann. Intern. Med..

[18] Yarnell P.R., Chu N.S. (1975). Focal seizures and aminophylline. Neurology.

[19] Boison D. (2011). Methylxanthines, seizures, and excitotoxicity. Handb. Exp. Pharmacol..

[20] Yokoyama H., Onodera K., Yagi T., Iinuma K. (1997). Therapeutic doses of theophylline exert proconvulsant effects in developing mice. Brain Dev..

[21] Wlaz P., Rolinski Z., Kleinroki Z., Czuczwar S.J. (1994). Influence of chronic aminophylline on antielctroshock activity of diazepam and aminophylline induced convulsions. Pharmacol. Biochem. Behav..

[22] Sarro A.D., Grasso S., Zappalà M., Nava F., Sarro G.D. (1997). Convulsant effects of some xanthine derivatives in genetically epilepsy-prone rats. Naunyn Schmied. Arch. Pharmacol..

[23] Czuczwar S.J., Kleinrok Z. (1990). Modulation of the protective efficacu of common antiepileptic drugs by xanthine derivatives: Implications for the clinical use of xanthines in epileptic patients. Pharmacol. Res..

[24] Guarienti M., Giacopuzzi E., Gianoncelli A., Sigala S., Spano P., Pecorelli S., Pani L., Memo M. (2015). Computational and functional analysis of biopharmaceutical drugs in zebrafish: Erythropoietin as a test model. Pharmacol. Res..

[25] Gianoncelli A., Bonini S.A., Bertuzzi M., Guarienti M., Vezzoli S., Kumar R., Delbarba A., Mastinu A., Sigala S., Spano P. (2015). An integrated approach for a structural and functional evaluation of biosimilars: Implications for erythropoietin. BioDrugs.

[26] Ašmonaitė G., Boyer S., Souza K., Wassmur B., Sturve J. (2016). Behavioural toxicity assessment of silver ions and nanoparticles on zebrafish using a locomotion profiling approach. Aquat. Toxicol..

[27] Strähle U., Scholz S., Geisler R., Greiner P., Hollert H., Rastegar S., Schumacher A., Selderslaghs I., Weiss C., Witters H. (2012). Zebrafish embryos as an alternative to animal experiments—A commentary on the definition of the onset of protected life stages in animal welfare regulations. Reprod. Toxicol..

[28] Olivares C.I., Field J.A., Simonich M., Tanguay R.L., Sierra-Alvarez R. (2016). Arsenic (III, V), indium (III), and gallium (III) toxicity to zebrafish embryos using a high-throughput multi-endpoint in vivo developmental and behavioral assay. Chemosphere.

[29] Gunnarsson L., Jauhiainen A., Kristiansson E., Nerman O., Larsson D.G. (2008). Evolutionary conservation of human drug targets in organisms used for environmental risk assessments. Environ. Sci. Technol..

[30] Howe K., Clark M.D., Torroja C.F., Torrance J., Berthelot C., Muffato M., Collins J.E., Humphray S., McLaren K., Matthews L. (2013). The zebrafish reference genome sequence and its relationship to the human genome. Nature.

[31] Lawrence C. (2007). The husbandry of zebrafish (*Danio rerio*): A review. Aquaculture.

[32] Kalueff A.V., Stewart A.M., Gerlai R. (2014). Zebrafish as an emerging model for studying complex brain disorders. Trends Pharmacol. Sci..

[33] Ali S., Champagne D.L., Richardson M.K. (2012). Behavioral profiling of zebrafish embryos exposed to a panel of 60 water-soluble compounds. Behav. Brain Res..

[34] Stewart A.M., Desmond D., Kyzar E., Gaikwad S., Roth A., Riehl R., Collins C., Monnig L., Green J., Kalueff A.V. (2012). Perspectives of zebrafish models of epilepsy: What, how and where next?. Brain Res. Bull..

[35] Kalueff A.V., Gebhardt M., Stewart A.M., Cachat J.M., Brimmer M., Chawla J.S., Craddock C., Kyzar E.J., Roth A., Landsman S. (2013). Towards a comprehensive catalog of zebrafish behavior 1.0 and beyond. Zebrafish.

[36] Baraban S.C., Taylor M.R., Castro P.A., Baier H. (2005). Pentylenetetrazole induced changes in zebrafish behavior, neural activity and c-fos expression. Neuroscience.

[37] Afrikanova T., Serruys A.-S.K., Buenafe O.E.M., Clinckers R., Smolders I., de Witte P.A.M., Crawford A.D., Esguerra C.V. (2013). Validation of the zebrafish pentylenetetrazol seizure model: Locomotor versus electrographic responses to antiepileptic drugs. PLoS ONE.

[38] Organisation for Economic Co-operation and Development (OECD) (2013). Test Guideline 236. Guideline for Testing of Chemicals, Fish Embryo Acute toxicity (Fet) Test.

[39] Lammer E., Carr G.J., Wendler K., Rawlings J.M., Belanger S.E., Braunbeck T. (2009). Is the Fish Embryo Toxicity test (FET) with the zebrafish (*Danio rerio*) a potential alternative for the fish acute toxicity test?. Comp. Biochem. Physiol. C Toxicol. Pharmacol..

[40] McKeown K.A., Downes G.B., Hutson L.D. (2009). Modular laboratory exercises to analyze the development of zebrafish motor behavior. Zebrafish.

[41] Kunkel L.M., Goody M.F., Kelly M.W., Reynolds C.J., Khalil A., Crawford B.D., Henry C.A. (2012). NAD+ biosynthesis ameliorates a zebrafish model of muscular dystrophy. PLoS Biol..

[42] Saint-Amant L., Drapeau P. (1998). Time course of the development of motor behaviors in the zebrafish embryo. J. Neurobiol..

[43] Goody M.F., Henry C.A. (2013). Motility assay for zebrafish embryos. Bio Protocol.

[44] Selderslaghs I.W.T., Hooyberghs J., Blust R., Witters H.E. (2013). Assessment of the developmental neurotoxicity of compounds by measuring locomotor activity in zebrafish embryos and larvae. Neurotoxicol. Teratol..

[45] Parsons W.J., Ramkumar V., Stiles G.L. (1987). Isobutylmethyixanthine stimulates adenylate cyclase by blocking the inhibitory regulatory protein, Gi. Mol. Pharmacol..

[46] Kukovetz W.R., Pöch G., Holzmann S. (1983). Overadditive synergism between theophylline, diprophylline and proxyphylline in tracheal smooth muscle relaxation. Arzneimittelforschung.

[47] Baggott M.J., Childs E., Hart A.B., de Bruin E., Palmer A.A., Wilkinson J.E., de Wit H. (2013). Psychopharmacology of theobromine in healthy volunteers. Psychopharmacology.

[48] Guarienti M., Gianoncelli A., Bontempi E., Moscoso Cardozo S., Borgese L., Zizioli D., Mitola S., Depero L.E., Presta M. (2014). Biosafe inertization of municipal solid waste incinerator residues by cosmos technology. J. Hazard. Mater..

[49] Raldua D., Pina B. (2014). In vivo zebrafish assays for analyzing drug toxicity. Expert Opin. Drug Metab. Toxicol..

[50] Mumford G.K., Evans S.M., Kaminski B.J., Preston K.L., Sannerud C.A., Silverman K., Griffiths R.R. (1994). Discriminative stimulus and subjective efects of theobromine and caffeine in human. Psychopharmacology.

[51] Tran T.C., Sneed B., Haider J., Blavo D., White A., Aiyejorun T., Baranowski T.C., Rubinstein A.L., Doan T.N., Dingledine R. (2007). Automated, quantitative screening assay for antiangiogenic compounds using transgenic zebrafish. Cancer Res..

[52] Lantz-McPeak S., Guo X., Cuevas E., Dumas M., Newport G.D., Ali S.F., Paule M.G., Kanungo J. (2015). Developmental toxicity assay using high content screening of zebrafish embryos. J. Appl. Toxicol..

[53] Antkiewicz D.S. (2005). Heart malformation is an early response to tcdd in embryonic zebrafish. Toxicol. Sci..

[54] Chemidplus: A Toxnet Database. https://chem.sis.nlm.nih.gov/chemidplus/chemidlite.jsp.

[55] Chemidplus: A Toxnet Database. Substance Name: Aminophylline. https://chem.sis.nlm.nih.gov/chemidplus/rn/317-34-0.

[56] Chemidplus: A Toxnet Database. Substance Name: Caffeine. https://chem.sis.nlm.nih.gov/chemidplus/rn/58-08-2.

[57] Chemidplus: A Toxnet Database. Substance Name: Diprophylline. https://chem.sis.nlm.nih.gov/chemidplus/rn/479-18-5.

[58] Chemidplus: A Toxnet Database. Substance Name: Doxofylline. https://chem.sis.nlm.nih.gov/chemidplus/rn/69975-86-6.

[59] Chemidplus: A Toxnet Database. Substance Name: Etophylline. https://chem.sis.nlm.nih.gov/chemidplus/rn/519-37-9.

[60] Chemidplus: A Toxnet Database. Substance Name: 1-Methyl-3-isobutylxanthine. https://chem.sis.nlm.nih.gov/chemidplus/rn/28822-58-4.

[61] Chemidplus: A Toxnet Database. Substance Name: Pentoxifylline. https://chem.sis.nlm.nih.gov/chemidplus/rn/6493-05-6.

[62] Chemidplus: A Toxnet Database. Substance Name: Theobromine. https://chem.sis.nlm.nih.gov/chemidplus/rn/83-67-0.

[63] Chemidplus: A Toxnet Database. Substance Name: Theophylline. https://chem.sis.nlm.nih.gov/chemidplus/rn/58-55-9.

[64] Knöbel M., Busser F.J., Rico-Rico A., Kramer N.I., Hermens J.L., Hafner C., Tanneberger K., Schirmer K., Scholz S. (2012). Predicting adult fish acute lethality with the zebrafish embryo: Relevance of test duration, endpoints, compound properties, and exposure concentration analysis. Environ. Sci. Technol..

[65] Nagel R. (2002). DarT: The embryo test with the Zebrafish *Danio rerio*—A general model in ecotoxicology and toxicology. ALTEX.

[66] Klüver N., König M., Ortmann J., Massei R., Paschke A., Kühne R., Scholz S. (2015). Fish embryo toxicity test: Identification of compounds with weak toxicity and analysis of behavioral effects to improve prediction of acute toxicity for neurotoxic compounds. Environ. Sci. Technol..

[67] Selderslaghs I.W., van Rompay A.R., de Coen W., Witters H.E. (2009). Development of a screening assay to identify teratogenic and embryotoxic chemicals using the zebrafish embryo. Reprod. Toxicol..

[68] Belanger S.E., Rawlings J.M., Carr G.J. (2013). Use of fish embryo toxicity tests for the prediction of acute fish toxicity to chemicals. Environ. Toxicol. Chem..

[69] Westerfield M. (2000). A Guide for the Laboratory Use of Zebrafish (Danio Rerio).

