# Supplementary material for: Zebrafish Embryo as an In Vivo Model for Behavioral and Pharmacological Characterization of Methylxanthine Drugs
Source: Int J Mol Sci. 2017 Mar 9;18(3):596. doi: 10.3390/ijms18030596 (PMC5372612; doi:10.3390/ijms18030596)
Supplement: Supplementary file 1 [file ijms-18-00596-s001.zip › ijms-180128-suppl-Table S4.pdf]

Supplementary S4. Specific endpoints with the corresponding timepoint and concentration at which they were first observed during the FET-test.

Compound	First Affected Endpoint	Timepoint (hpf)	Concentration (mg/L)
Aminophylline	Movement	24	250
Caffeine	Movement	24	90
Diprophylline	Heartbeat	48	4000
Doxofylline	Movement	24	250
Etofylline	Heartbeat; blood circulation	48	200
IBMX	Movement; detachment of tail	24	50
Pentoxifylline	Movement	24	100
Theobromine	Heartbeat	48	100
Theophylline	Movement	24	100
